# Covid-19 outbreak and beyond: a retrospect on the information content of short-time workers for GDP now- and forecasting

**DOI:** 10.1186/s41937-023-00106-x

**Published:** 2023-01-31

**Authors:** Sylvia Kaufmann

**Affiliations:** 1 Study Center Gerzensee, Foundation of the Swiss National Bank, Dorfstrasse 2, 3115 Gerzensee, Switzerland; 2 Faculty of Business and Economics, University of Basel, Peter Merian-Weg 6, 4002 Basel, Switzerland

**Keywords:** Bayesian analysis, Covid-19, Pseudo-real-time, Ordinances, SECO, KOF, C32, C53, E23, E27

## Abstract

We document whether a simple, univariate model for quarterly GDP growth is able to deliver forecasts of yearly GDP growth in a crisis period like the Covid-19 pandemic, which may serve cross-checking forecasts obtained from elaborate and expert models used by forecasting institutions. We include shocks to the log number of short-time workers as timely available current-quarter indicator. Yearly GDP growth forecasts serve cross-checking, in particular at the outbreak of the pandemic.

## Introduction

At the beginning of 2020, the Covid-19 pandemic spread in Europe and led to unprecedented disruptions in social and economic activities. A most pressing issue by that time was to evaluate the current or immediate economic stance and obtain a forecast of the intermediate outlook. Measurements of usual monthly (survey) indicators used in Swiss GDP now- and forecasting virtually became outdated overnight. The monthly release calendar proved to be too low frequency to obtain timely updates of the economic stance. There was a quest for indicators related to actual productive activity released at a higher frequency, on a weekly or even daily basis. A daily indicator, a fever or *f*-curve, was developed in Burri and Kaufmann (2020). The *f*-curve corresponds to the first principle component extracted from publicly available financial market data and news sentiment indicators built from articles of major Swiss journals. The *f*-curve turns out to highly correlate with survey indicators of Swiss economic activity.^1^ Lengwiler (2020) used electricity consumption available from Swissgrid, a monthly series and a daily series aggregated from a 15-minute frequency. The extracted electricity gap showed a significant negative shift on March 13, 2020. Eckert et al. (2020) set up a mixed-frequency factor model for a large dataset including additional series highly correlated with current production, available at a weekly or daily frequency. The extracted factor identifies well as a weekly series for GDP growth. Likewise, Wegmüller et al. (2023) extract a weekly economic activity index from selected nine daily or weekly series, carefully adjusted for calendar and seasonal effects prior extraction. The indicator highly correlates with and improves now-casting of GDP growth. Finally, Brown et al. (2020) and Becerra et al. (2020) exploited increased data availability and developed platforms that visualize in real time, respectively, individual payments data or compiled economic indicators based on Google search.^2^

Compared with these studies, which focus on extracting the actual economic stance at a daily or weekly frequency or now-casting quarterly GDP growth, this paper uses a simple model to provide forecasts of current- and following-year yearly GDP growth during a crisis. These forecasts are cross-checked with forecasts published by two Swiss forecasting institutions, the State Secretariat of Economic Affairs (SECO) and KOF Swiss Economic Institute (KOF). In the parent paper to the present one, Kaufmann (2020) (KA20 in the following) exploited the timely available number of short-time workers to obtain a now- and forecast of quarterly GDP growth at the outbreak of the Covid-19 pandemic. In a first univariate auto-regression, the log number of monthly short-time workers was purged from the systematic component, to obtain the shocks or innovations to the series. Monthly innovations were cumulated to quarterly shocks, which entered contemporaneously and with lags a second univariate regression fitted to quarterly GDP growth. It turned out that shocks explained an additional 24% of variation in GDP growth, and the model forecasted well the decline in quarterly level GDP during the financial crisis. At the time, the model forecasted a maximum decline in quarterly GDP of − 5.7%, with the highest forecast density interval (HFDI) of − 9.5% to − 2.9%. The forecast was quite in line with those published by forecast institutions in Switzerland, like SECO or KOF.

The forecasts computed in KA20 by May 2020 were based on a data download from the SECO webpage including numbers on short-time workers up to January 2020. This series was completed with numbers of short-time workers pre-registered for short-time work from February to April 2020, obtained by e-mail from SECO. Data vintages on short-time workers as well as numbers of workers pre-registered for short-time work are unavailable, unfortunately. Therefore, for the present paper, we build a pseudo-real-time data bank based on numbers of workers with settled payments after 30 to 210 days following the settlement period.^3^ For GDP, we use the real-time dataset available on the SECO webpage (Indergand and Leist, 2014).

We end the introduction by providing some historical background. Section 2 then presents the data and the building of data vintages. Section 3 outlines the econometric approach, presents the model and forecasting equations, and details the forecasting procedure. Section 4 discusses the results and compares forecasts to institutional forecasts. Section 5 concludes.

*Some historical background* This follow-up paper is motivated by the fact that the pandemic has lasted much longer than first expected in 2020. To curb the first wave of infections, most countries followed lockdown strategies, imposing very strict social distancing measures. The benefit of reducing new infections to a very low number within two to three months came at high economic and social costs. Undoubtedly, the imposed measures saved lives and prevented the health system, and eventually the economy, to collapse (Gatti and Retali, 2021). On the other hand, early studies also report (negative) distributional effects, whereby generally households at the lower-end of the income distribution were affected more heavily by the pandemic in various dimensions like health, education and income (Martínez et al. 2021; Fuchs-Schündeln et al. 2022b, a). Moreover, even very strict and longer-lasting lockdown strategies proved unsuccessful in preventing resurgent infection waves. Switzerland experienced five waves, the fifth during Spring 2022 with a record-high incidence exceeding 4000 [compared to roughly 160 (300) in April 2020 (2021)].^4^ Although more infectious, mutations of the Sars-Cov-2 virus proved less aggressive. Against the background of an increasing share of population vaccinated or recovered, the virus eventually has become endemic (Bundesrat, 2022).

In the course of the pandemic, Switzerland never again introduced such strict measures as in the first half of 2020.^5^ Subject to quarantining and testing rules, persons could always move freely. Beginning 2021, re-installed federal restrictions concerned mainly restaurants and recreational businesses, without imposing a complete shutdown, however. For example, while indoor dining was prohibited, restaurants were allowed to provide take-away and later on outdoor dining services. The maximum number of persons per group, or imposed indoor social distancing measures were successively relaxed during Spring 2021. To counteract the negative effect of ongoing cantonal and re-installed federal restrictions, the Federal Government prolonged or reactivated some of the simplified administrative procedures installed in Spring 2020 to apply for and obtain short-time work benefits, like cancelling the waiting time, offering summary settlement, extending the eligibility period, or disregarding earnings from secondary employment.^6^ After the unprecedented increase to 1.4 million short-time workers in April 2020,^7^ numbers decreased to a quarter million (254,000) in October. In November, the number of short-time workers started increasing again to reach over half a million (524,000) in February 2021. Since then, numbers have decreased, reaching 48,000 in October 2021.

## Data and data vintages

### Data

**Fig. 1:**
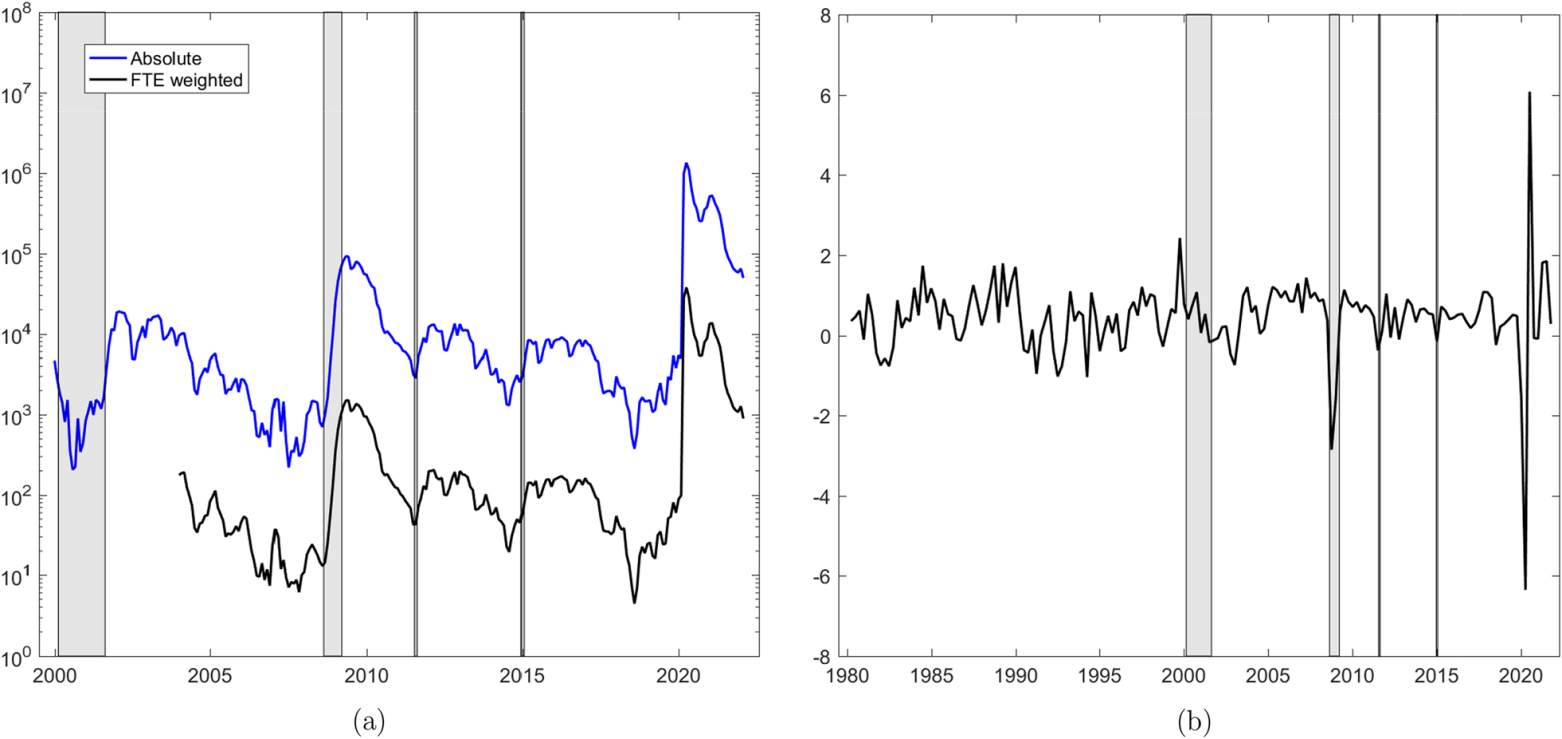
Time series. **a** Short-time workers (download May 25, 2022), total of absolute numbers across sectors (Absolute) and total across sectors weighted by division-specific full-time-equivalent employment (FTE weighted). Monthly frequency, logarithmic scale. **b** Real GDP growth (vintage 22Q1), quarterly frequency, percentage scale. Gray bars highlight the dotcom and financial crises, the introduction and discontinuation of the euro-Swiss franc floor. Notes: Short-time workers, concatenated data: Published pdf-file (https://www.seco.admin.ch/seco/de/home/Arbeit/Arbeitslosenversicherung/leistungen/kurzarbeitsentschaedigung.html) as of May 1, 2020, January 2000 to December 2003; download (https://www.amstat.ch/MicroStrategy/servlet/mstrWeb) as of May 25, 2022, January 2004 to February 2022. Division-specific full-time equivalent employment: Data for 2019, obtained from FSO by e-mail as of June 20, 2022. GDP growth: Vintage 22Q1, Download (https://www.seco.admin.ch/seco/de/home/wirtschaftslage—wirtschaftspolitik/Wirtschaftslage/bip-quartalsschaetzungen-/concepts–en–.html) as of May 28, 2022. The shaded dotcom crisis spans the burst of the bubble in March 2000 up to the Enron scandal in October 2001. The shaded area of the financial crisis spans from September 2008 (Lehman Brothers insolvency) to April 2009

Figure 1, Panel (a), plots the monthly number of short-time workers on a logarithmic scale.^8^ The data download from www.amstat.ch as of May 25, 2022, covers the period January 2004 to February 2022. The series is concatenated with data from a pdf file published on the website of the State Secretariat of Economic Affairs (SECO), to obtain a sample starting in January 2000. We see the unprecedented increase to 1.4 (26.9% of working population) and 1.1 million (21.2%) in, respectively, April and May 2020. As Covid-related restrictions successively were relaxed from June 2020 onwards, numbers decreased to roughly 254,000 in October 2020. To curb the second wave of infections starting in Fall 2020, restrictions were re-installed first canton-wise, and ultimately country-wide towards the end of 2020. Against the background of ongoing restrictions, the number of short-time workers increased again to reach 524,000 in February 2021. Since April 2021, the number of short-time workers has decreased continuously to around 50,000 in February 2022, a level comparable to the peak during the financial crisis.

Extreme volatility in short-time workers transmits directly into productive capacity. Panel (b) of Fig. 1 plots quarterly GDP growth (quarter-on-quarter, in percentage terms), the data vintage in the first quarter 2022 (22Q1), downloaded from the SECO website as of May 28 2022. The drop by more than 6% in the second quarter 2020 is unprecedented, more than twice as large as the drop at the onset of the financial crisis. Unlike the financial crisis shock, the Covid-19 shock is expected to have a transitory effect on level GDP. The large negative growth rate in the second quarter has been offset by an equally large rebound in the third quarter of 2020. In retrospect, the negative effect of the Covid-19 outbreak has been much milder than expected early in 2020. Quarterly GDP figures published by SECO imply a decrease in real GDP by 2.5% for 2020,^9^, whereas in April (May) 2020 SECO (KOF) forecasted GDP to drop by 6.7% (5.5%).

**Fig. 2:**
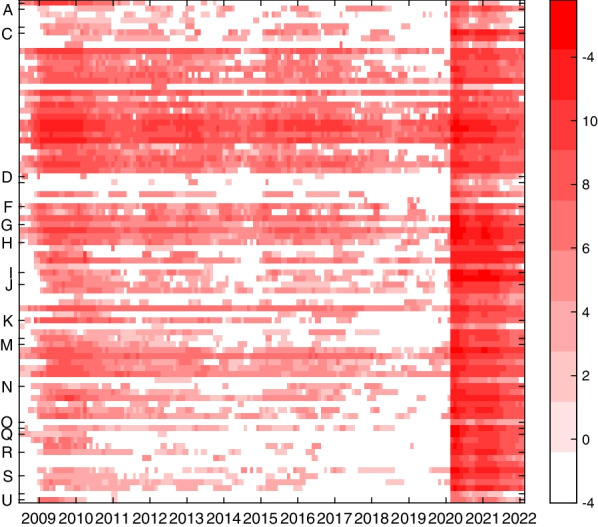
Sectoral time series. Monthly frequency, July 2008–February 2022, heat plot of log short-time workers (0 is transformed to $\log (e^{-4})$), see Federal Statistical Office (2008) for a list of activities associated to Sections A to U. Notes: Download (https://www.amstat.ch/MicroStrategy/servlet/mstrWeb) as of May 25, 2022, January 2004 to February 2022

Figure 2 presents a heatmap of log short-time workers at the two-digit, i.e. division-specific level of the General Classification of Economic Activities (NOGA) over the period July 2008 to February 2022, where 0 is transformed to $\log (e^{-4})$. Unprecedented, in March 2020 workers in all divisions went persistently into short-time work, whereas up to February 2020, many divisions had recorded only few short-time workers at irregular intervals.^10^ In Fig. 1, Panel (a), we plot the sum of short-time workers across divisions weighted by division-specific full-time-equivalent employment in 2019.^11^ The series follows closely the sum of absolute numbers across divisions.^12^ This suggests that working with the absolute total of short-time workers reflects well the situation of the aggregate FTE equivalent series.

Obviously, high volatility in production renders now- and forecasting GDP growth during a crisis very difficult. When most sectors, and in particular the large service sector, of an economy face recurrently changes between regimes of tight and looser restrictions, current and future outcomes become less predictable. In the following, we assess whether the simple univariate model used in KA20 provides yearly GDP forecasts that could serve cross-checking forecasts obtained from more elaborate and expert models.

### Data vintages

**Fig. 3:**
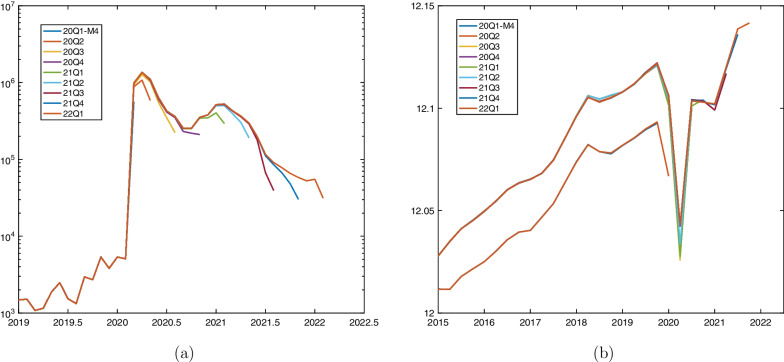
Time series vintages. **a** Short-time workers. Monthly frequency, logarithmic scale, observed up to March for forecasting start date 2020Q1-M4 (end April) and end of quarter’s second month for all other forecasting start dates. **b** Log real GDP, quarterly frequency, observed up to the lagged quarter of the forecasting start date (also 2019Q4 for start date 2020Q1-M4). Notes: Short-time workers, concatenated data: Published pdf-file (https://www.seco.admin.ch/seco/de/home/Arbeit/Arbeitslosenversicherung/leistungen/kurzarbeitsentschaedigung.html) as of May 1, 2020, January 2000–December 2003; download (https://www.amstat.ch/MicroStrategy/servlet/mstrWeb) as of May 25, 2022, January 2004 to February 2022. Vintages constructed from April 2020 to April 2022 using data on short-time workers with settled payments after one to seven months following the settlement period. GDP growth: Download (https://www.seco.admin.ch/seco/de/home/wirtschaftslage—wirtschaftspolitik/Wirtschaftslage/bip-quartalsschaetzungen-/concepts–en–.html) as of May 28, 2022

Data displayed in Fig. 1 represent the last vintage available, while for the forecasting exercise, we will use the data vintages plotted in Fig. 3, to reconstruct as closely as possible the situation faced by forecasters in real time.

Numbers of short-time workers are published with a lag of three months, and revised with each release as payments are settled, the revisions being smaller for months lying further in the past. Unfortunately, no history of vintages is available. And, even if it would be available, current-quarter information on short-time workers would not be available for a nowcast of GDP growth. For producing the forecasts in KA20, we received from SECO numbers on short-time workers pre-registered for current-quarter months. Vintages of pre-registered numbers are not available, either. However, by May 25 2022, SECO provided vintages of numbers of short-time workers with settled payments after 30 (one month) to 210 days (seven months) following the settlement period.^13^ Therefore, month-specific vintage numbers increase over time, and after seven months, do not change substantially anymore. The vintages are provided for March 2020 to February 2022. We interpret the vintages as follows. For March 2020, a first number of short-time workers with settled payments after 30 days of the settlement period (March 2020) would be available by the end of April. In May 2022, the most recent number of short-time workers for February 2022 would be available by the end of April (60 days after the settlement period). The numbers of short-time workers with settled payments more than 210 days after the settlement period are set to numbers downloaded as of May 25, 2022. The vintages used for forecasting are plotted in Fig. 3, Panel (a). We observe that typically, numbers of early vintages lie below the final-vintage data. Also, these vintage data lie presumably below numbers of short-time workers pre-registered for month-specific settlement periods. For example, while 1.6 million workers were pre-registered for short-time work in March 2020, the end-vintage reports that roughly 985,000 only obtained short-time work compensation.

For GDP vintages, we use the real-time dataset provided on the website of SECO (Indergand and Leist, 2014). The vintages are plotted in Panel (b) of Fig. 3. The vintage 2020Q3, publishing a first release for GDP in the second quarter of 2020, shows a simultaneous level adjustment without substantial dynamic changes. Except for 2020Q2 figures, published GDP figures are not revised substantially, either.

## Econometric approach

### Model and forecasting equations

We first fit an autoregressive process to the log number of short-time workers $n_t^s$

1 $$\begin{aligned} n_t^{s}=\mu ^s_n+ \varphi _1 n^s_{t-1}+\ldots + \varphi _l n^s_{t-l}+\nu ^s_{t},\ \nu ^s_t\sim {\text {i.i.d.}}N(0,\delta ^2) \end{aligned}$$

where $t=1,\dots , T_n$ is a monthly time index. Cumulate within-quarter monthly shocks to obtain a quarterly series of shocks to short-time workers, $\nu ^q_{t}=\sum _{j=0}^2 \nu ^s_{t-j}$. This series captures the unsystematic or news component in short-time workers. Included along with four lags in an univariate autoregressive regression fitted to quarterly GDP, these shocks explained additional 24% of data variation (KA20). However, estimates suggest that lags of shocks in short-time workers have no marginal effect on current-quarter GDP growth, see Equation (8) in KA20, where the highest posterior density intervals are roughly centred at zero. In the following analysis, we take into account that unusually large shocks nevertheless may have a more persistent effect on GDP growth in the following periods. We proceed by cumulating shocks in short-time workers up to *m* lagged quarters, $\nu ^q_{t,m}=\sum _{j=0}^m \nu ^q_{t-j}$, and add this information to explain variation in current-quarter GDP growth $y_t$:

2 $$\begin{aligned} y_{t} & = \mu _{y} + \theta \nu _{{t,m}}^{q} + \phi _{1} y_{{t - 1}} + \ldots + \phi _{p} y_{{t - p}}\\ & \quad + \sum\limits_{{j = 1}}^{3} {\psi _{j} } D_{{jt}} + \varepsilon _{t} ,\;\varepsilon _{t} \sim{\text{i.i.d}}N(0,\sigma ^{2} ) \\ \end{aligned}$$

where $t=1,\ldots , T$ is a quarterly time index, and $D_{jt}$ represent quarterly dummies. Equation (2) comes close to a bridge equation (Foroni and Marcellino, 2013; Stock and Watson, 2002), except that the high-frequency covariate entering the equation is the innovation or residual of estimated Eq. (1). The approach is also in the spirit of Romer and Romer (1989, 2004), who regressed output and inflation on monetary policy shocks to evaluate monetary policy effects.

Specification (2) provides the basis to forecast quarterly GDP growth from quarter *F* onwards, $F>T$. For posterior inference, *m* is specified such that first-quarter forecasts $y_F$ include the information of shocks to short-time workers cumulated since the outbreak of the pandemic, i.e. since the first quarter 2020, see details in the following subsection. As motivated above, cumulating shocks in short-time workers since the outbreak of the pandemic takes into account the potential (decreasing) lagged effect of the initial unprecedented shock in March 2020 on current-quarter GDP forecast. Of course, this procedure is pandemic- or crisis-specific, and *m* may remain fixed after the economy is deemed recovering again.^14^

Posterior inference of Eqs. (1) and (2) is obtained by Bayesian Markov chain Monte Carlo methods, see the sampling steps described in Subsection 2.3 of KA20. The mean across draws of residuals $\nu ^s_t$ flow into $\nu ^q_{t,m}$.

We obtain dynamic forecasts and the forecast distribution by a posterior predictive analysis, using the posterior sample of parameters:

3 $$\begin{aligned} y_{{F + h}} & = \hat{\mu }_{y} + \hat{\theta }\nu _{{F + h,m}}^{f} + \hat{\phi }_{1} y_{{F + h - 1}} + \hat{\phi }_{p} y_{{F + h - p}} \\ & \quad + \sum\limits_{{j = 1}}^{3} {\hat{\psi }_{j} } D_{{jt}} + \hat{\varepsilon }_{{F + h}} ,\;h = 0, \ldots ,H \\ \end{aligned}$$

where *F* is the starting quarter of the forecast window, and $y_{F+h-j}$ is observed if $F+h-j< F$. The hat indicates that a forecast series (or projection) is obtained for each posterior draw of parameters; note that we incorporate model uncertainty $\hat{\varepsilon }_{F+h}$, drawing from $N(0,\hat{\sigma }^2)$. Shocks $\nu ^f_{F+h,m}=\sum _{j=0}^m \nu ^q_{F+h-j}$ cumulate $\nu ^s_{t}$ for $t<T_n$, and $\nu ^s_t=0$ for $t>T_n$.

### Forecasting procedure

In the following pseudo-real-time exercise, we produce dynamic quarterly GDP growth forecasts up to horizon *H* based on Eq. (3), starting with a so-called nowcast for quarter *F*, where *F* runs from the first quarter 2020 (produced by the end of April) to the first quarter of 2022, $F=2020Q1\text {-}M4, 2020Q2, \dots , 2022Q1$. The first forecast series is produced by the end of April ($2020Q1\text {-}M4$), while the remaining are produced by the end of the respective quarter’s third month. Using these quarterly forecasts, we derive implied forecasts for yearly GDP growth rates.

During a highly volatile crisis, we may have to make specific choices as regards the sample to use for estimating Eqs. (1 and 2). In Panel (a) of Fig. 1, we observe that the Covid outbreak manifests as a one-time, huge shock in short-time workers, without dramatically changing autoregressive dynamics. Therefore, we expect posterior inference of Eq. (1) to remain quite stable, even if the sample end $T_n$ extends beyond the first quarter 2020. On the other hand, Panel (b) shows that the volatility in GDP growth remains persistently higher after the Covid outbreak and comes along with a considerable change in dynamics when compared with historical figures. At the outbreak of the pandemic, economic forecasting institutions expected GDP to recover fast, growth dynamics to remain volatile, though. In this situation, estimates of Eq. (2), in particular estimates of autoregressive coefficients, would be expected to highly depend on the sample window chosen. Extending *T* beyond the first quarter 2020, most likely would reverse the sign of an usually positively estimated first-order autoregressive coefficient. Ultimately, this would induce unusual dynamics into forecasts starting in periods following the first quarter 2020. Against the background of these considerations, we proceed as follows.

**Fig. 4:**
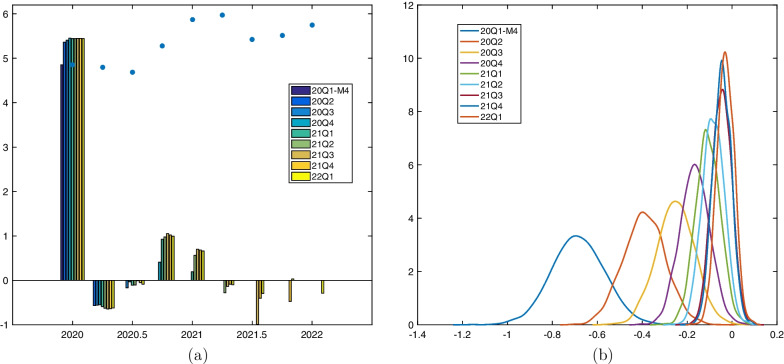
Log short-time workers. **a** Mean in-sample one-step ahead forecast error. Expanding sample period: April 2000–March 2020 for forecasting start date $F=20Q1\text {-}M4$ and April 2000 to quarter’s second month for starting forecast quarter $F=20Q2,\dots ,22Q1$. The dots represent cumulated shocks up to forecasting start date (the sum across equally colored bars). **b** Posterior distribution of $\theta$. Sample period: First quarter 2000 $+ \max (p,m)$ to fourth quarter 2019

To obtain the series of shocks $\nu ^s_t$, we estimate Eq. (1) applying an expanding window up to including the most recent observation $n^s_t$ available by the forecast starting date, i.e. up to the first release for March 2020 when $F=2020Q1\text {-}M4$, and to the respective quarter’s second month when $F=2020Q2,\dots , 2022Q1$. Based on results in KA20, we set $l=3$. Figure 4, Panel (a), plots the mean in-sample one-step ahead forecast errors, cumulated within-quarter to $\nu ^q_t$, obtained when expanding the sample size $T_n$ up to the most recent available observation for each forecast window. As expected, expanding the estimation window beyond the first quarter of 2020 does not have a large effect on estimates, and shocks are mainly revised between the first and second release of quarter-specific observations only. For example, the 2020*Q*1 shock compiled for $F=2020Q1\text {-}M4$ is revised upwards once for $F=2020Q2$ and remains at the same level for all following forecast windows.

As motivated above, we estimate Eq. (2) based on a fixed window, i.e. the sample end *T* is always the fourth quarter of 2019. According to results in KA20, we set $p=2$. As we move *F* in Eq. (3) further into 2020 and 2021, we expand the window over which we accumulate shocks, $m=F-T-1$, in order to estimate the marginal effect of $\nu ^q_{t,m}$ on current-quarter GDP growth. The nowcast $y^f_F$ thus includes the effect of $\nu ^q_{F,F-T-1}$, i.e. the effect of all shocks cumulated since the first quarter 2020. The dots in Panel (a) of Fig. 1 represent $\nu ^q_{F,F-T-1}$ (the sum across equally colored bars), and Panel (b) plots the posterior distribution of $\theta$ conditional on specified shocks $\nu ^q_{t,F-T-1}$, $F=2020Q1\text {-}M4,\dots ,2022Q1$. The marginal effect of shocks decreases as *F* moves further ahead. The result confirms that Eq. (2) with a specification of *m* depending on *F* is able to incorporate the notion that the marginal effect of a (large) shock cumulated into $\nu ^q_{t,m}$ diminishes the further back in time it occurred.

## Forecasts and comparison with institutional forecasts

**Fig. 5:**
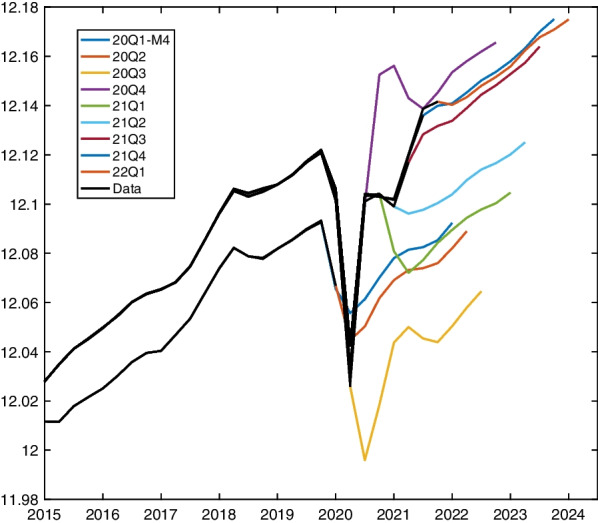
Out-of-sample implied quarterly log GDP forecast

Using Eq. (3), we produce forecasts of quarterly GDP growth over sequential forecast windows starting in $F=2020Q1\text {-}M4,\dots ,2022Q1$ and ending eight quarters ahead, $H=8$. The implied mean forecasts for log-level quarterly GDP are plotted in Fig. 5. All projections starting in a quarter of 2020 are highly volatile, whereby first-period forecasts inherit previous periods’ direction before mean-reverting to the growth pattern implied by the autoregressive process. The absence of new substantial shocks after 2020, renders projections smoother, reflecting mainly the autoregressive process estimated for the pre-crisis period.

**Table 1:** Yearly GDP growth forecasts and releases

	Date	2020	2021	2022	Date	2020$^*$	2021	2022	2023
*State Secretariat of Economic Affairs (2020a-2020e, 2021a-2021d, 2022), real time*									
	19/03/20	− 1.3	3.3		11/03/21		3.2	3.5	
	23/04/20	− 6.7	5.2						
	16/06/20	− 6.2	5.3		15/06/21		3.8	3.5	
	12/10/20	− 3.8	4.2		16/09/21		3.4	3.2	
	15/12/20	− 3.3	3.2	3.3	09/12/21		3.5	3.2	1.7
					14/03/22			3.0	1.7
*KOF Swiss Economic Institute (2020a-2020e, 2021a-2021d, 2022), real time*									
	17/03/20	0.3	1.4		25/03/21		3.0	2.8	
	15/05/20	− 5.5	5.4						
	16/06/20	− 5.1	4.3		22/06/21		4.0	2.8	
	22/10/20	− 3.6	3.2	2.4	06/10/21		3.2	3.6	1.5
	15/12/20	− 3.5	3.2	2.6	16/12/21		3.6	3.0	2.1
					23/03/22			3.0	2.0
*KA20, real-time, pre-registered data*									
	30/04/20	− 4.1	2.7						
		(− 6.5,− 1.5)	(− 0.1,5.4)						
*Short-time workers model, Equation (3), pseudo-real-time, settled data*									
	30/04/20	− 2.4	1.9		31/03/21	− 3.0	− 0.5	1.7	
		(− 4.1,− 0.7)	(0.4,3.4)				(− 3.6,2.7)	(− 0.3,3.8)	
	30/06/20	− 3.2	1.7		30/06/21	− 2.8	1.1	1.3	
		(− 4.3,− 2.0)	(0.1,3.4)				(− 0.1,2.4)	(− 0.9,3.4)	
	30/09/20	− 7.8	1.0		30/09/21	− 2.5	3.0	2.2	
		(− 9.3,− 6.4)	(− 2.3,3.8)				(2.1,3.7)	(0.1,4.6)	
	31/12/20	− 1.7	4.8	1.4	31/12/21	− 2.5	3.5	2.3	1.9
		(− 2.5,− 0.9)	(1.3,8.5)	(− 0.7,3.4)			(3.1,3.8)	(0.0,4.8)	(− 0.2,4.0)
					31/03/22	− 2.5	3.7	2.0	1.8
								(0.1,4.1)	(− 0.2,4.0)
*Federal Statistical Office (2021, 2022), release*									
					26/08/21	− 2.4			
					30/08/22		4.2		

*Growth rates recorded in data vintages

The third panel in Table 1 displays mean yearly GDP growth rates (95% HFDI) implied by these quarterly projections. For comparison, the table includes in the first and second panel forecasts released in real time by, respectively, SECO and KOF.^15^ Against the background of the unprecedented restrictions imposed in the first half of 2020, the forecasted negative GDP growth rates in the first half year were much larger than updates published in the second half year. While SECO and KOF forecasts diverged by more than 1% for 2020 in the first half-year, they aligned again within less than half a percentage point in the second half-year. The pandemic expected to be a one-year transitory event, GDP was predicted to strongly recover in 2021. Based on a real-time download completed with numbers on workers pre-registered for short-time work, forecasts published in KA20 implied a decrease (increase) in yearly GDP growth of 4.1% (2.7%) for 2020 (2021). The HFDI of ($-$6.5,$-$1.5) included all published forecasts of SECO and KOF, except for $-$6.7% published by SECO in April 2020.

In retrospect, the number of workers with settled payments for March (April) 2020 turned out to be lower than the number of pre-registered workers, roughly 985,0000 (1.4 million) versus 1.6 (1.9) million, respectively. The projections starting in the first quarter of 2020 including information by the end of April, forecast a mean decrease in GDP by 2.4% and a rebound by 1.9% in, respectively, 2020 and 2021. The mean forecast for 2020 happens to be identical to the decline of 2.4% released by the Federal Statistical Office in August 2021.^16^ Although the mean forecast for 2021 is lower than forecasts published during 2020 by SECO and KOF, the upper tail of the HFDI (0.4,3.4) nearly includes GDP growth rates published by SECO (3.5%) and KOF (3.6%) in December 2021.

The volatile projections starting in the third and fourth quarter 2020 translate into volatile forecasts for yearly GDP growth. The growth rates predicted by the end of September imply the Covid outbreak to have a permanent negative level effect on GDP, while in December growth rates forecast a rapid recovery to pre-crisis GDP levels in 2021 (see also projections 20*Q*3 and 20*Q*4 in Fig. 5). The December mean forecast for 2020 ($-$1.7%) is less pessimistic than figures published by SECO ($-$3.3%) and KOF ($-$3.5%), while the mean forecast for 2021 is 1.6 percentage points more optimistic.

Figures in Column 2020$^*$ report GDP growth rates for 2020 implied by consecutive data vintages. We observe that GDP figures were repeatedly revised, such that by the end of September yearly GDP growth for 2020 implied by quarterly data aligns with the release of the Federal Statistical Office (FSO) by the end of August 2021 ($-$2.4% for 2020). The revisions reflect the large uncertainty surrounding the effects of the pandemic outbreak.

The renewed increase in short-time workers in the fourth quarter 2020 translates into uncertain prospects for GDP growth at the beginning of 2021.^17^ The HFDI of ($-$3.6,2.7) shows that the mean forecast of $-$0.5% is highly uncertain, besides lying considerably below SECO and KOF forecasts of around 3.0%. Forecasts for 2021 align again broadly with SECO and KOF releases for projections starting in the second half of 2021. Forecasts lie at 3.5% or 3.6% (KOF) for 2021.^18^ Looking ahead, the model forecasts a moderate increase of around 2% in GDP for 2022, roughly 1% lower than forecasted by SECO and KOF. Nevertheless, the HFDI again encompasses these figures. For 2023, the model forecast aligns well with SECO and KOF releases.

## Conclusion

We document how a simple, univariate model for quarterly GDP growth rates fares during the years of the pandemic in predicting yearly GDP growth. The model includes shocks or news to the number of short-time workers as timely current-quarter available indicator. For the analysis, we build a pseudo-real-time dataset, using vintages on short-time workers with settled payments after 30 to 210 days following the settlement period. For GDP, we use a real-time dataset available on the SECO website.

Although less pessimistic, forecasts of yearly GDP growth implied by quarterly projections are in line with forecasts published by SECO and KOF at the outbreak of the crisis. In retrospect, conditional on effective numbers of short-time workers GDP would have been predicted to decline by 2.4% in 2020, a prediction that matches the decline released by the Federal Statistical Office in August 2021. Subsequent projections starting in 2020 turn out to be more if not too volatile. The projection starting in September imply the Covid outbreak to have a permanent negative level effect on GDP, while the one starting in December predicts a quick recovery to pre-crisis GDP level. Forecasts for 2021 improve and align to figures published by SECO and KOF in the second half year of 2021.

Obviously, an univariate model fitting quarterly GDP growth is too simple to deliver fully reliable projections. Nevertheless, results document that shocks in (log) short-time workers include valuable information to forecast GDP growth at the beginning of a crisis period like the pandemic outbreak in the first quarter 2020. Highly volatile, i.e. oscillating production rebounds call for dynamic model adjustments, for example in autoregressive dynamics, to obtain more stable forecasts as the crisis persists. However, as the effect of major shocks vanishes, projections imply forecasts of yearly GDP growth that are aligned with forecasts published by SECO and KOF.

## Data Availability

The datasets used and analysed during the current study are available from the corresponding author on reasonable request.

## Abbreviations

GDP: Gross domestic product
FSO: Federal Statistical Office
HFDI: Highest forecast density interval
KOF: KOF Swiss Economic Institute
SECO: State Secretariat of Economic Affairs

## Footnotes

1 The *f*-curve is published on a daily basis at https://github.com/dankaufmann/f-curve/

2 See monitoringconsumption.com or trendecon.org.

3 Bernhard Weber from SECO provided the data by e-mail (May 25, 2022).

4 Measured as 14-days incidence, per 100,000 population.

5 See Bundesrat (2020a) for the federal ordinance on measures to combat the Covid-19 epidemic. For an overview of changes in measures from April 27 to November 30 2020 see the table published online by the Federal Office of Public Health, https://www.bag.admin.ch/dam/bag/en/dokumente/mt/k-und-i/aktuelle-ausbrueche-pandemien/2019-nCoV/covid-19-tabelle-lockerung.pdf.download.pdf/Easing_of_measures_and_possible_next_steps.pdf, and for changes since December 1 to date https://www.bag.admin.ch/dam/bag/en/dokumente/mt/k-und-i/aktuelle-ausbrueche-pandemien/2019-nCoV/tabelle-aenderungen-massnahmen.pdf.download.pdf/ Changes_measures. pdf (Accessed as of January 31 2022).

6 See Bundesrat (2020b), for the version in place and past changes to the federal ordinance on measures related to the unemployment insurance during the Covid-19 epidemic (available in German, French and Italian). See Brühlhart et al. (2020) for an evaluation of businesses’ recourse to Covid-19 related financial support during 2020.

7 As of May 2020, 1.9 million employees or 36.7% of the working population were pre-registered for short-time work in April.

8 Here, I use the term workers rather than employees, as in March 2020, additional groups like self-employed and employed managing staff became as well eligible for short-time work benefits. As mentioned in KA20, the correlation between the number of short-time workers and lost working hours is.97. Therefore, the results should remain the same, irrespective of the indicator used.

9 In August 2021, the Federal Statistical Office, which reports GDP at the yearly frequency, published a drop in GDP by 2.4% for 2020 (https://www.bfs.admin.ch/bfs/de/home/statistiken/volkswirtschaft/volkswirtschaftliche-gesamtrechnung/bruttoinlandprodukt.html,accessed on January 31).

10 The only division which never recorded short-time work employees between July 2008 and February 2020 were Sewerage; Public administration and defence, compulsory social security; Gambling and betting activities. Divisions which always recorded some short-time workers during the same period were Printing and reproduction of recorded media; Manufacture of fabricated metal products, except machinery and equipment; Manufacture of computer, electronic and optical products; Manufacture of machinery and equipment; Other manufacturing; Wholesale trade, except of motor vehicles and motorcycles; Other professional, scientific and technical activities.

11 The weights were provided by the Federal Statistical Office (FSO) by e-mail (June 20, 2022).

12 Series are perfectly correlated in log levels (1.0) and highly in growth rates (0.97).

13 The data are available for research purposes from the author upon request.

14 Although we do not pursue the objective, an optimal *m* may be defined or derived based on some optimality criterion.

15 State Secretariat for Economic Affairs (2020a-2020e, 2021a-2021d, 2022), KOF Swiss Economic Institute (2020a-2020e, 2021a-2021d, 2022). Figures correspond to forecasts including large sports events. Forecasts released in March 2020 are reported for completeness.

16 Federal Statistical Office (2021).

17 Likewise, the decreasing trend in the daily fever curve of Burri and Kaufmann (2020) came temporarily to a halt around the turn of the year 2020 to 2021, see https://github.com/dankaufmann/f-curve/ (Accessed on January 31 2021).

18 By the time of drafting the second revision of the paper, the Federal Statistical Office (2022) had released a 4.2% growth in GDP for 2021.
